# Metabolism of Bile Salts in Mice Influences Spore Germination in *Clostridium difficile*

**DOI:** 10.1371/journal.pone.0008740

**Published:** 2010-01-15

**Authors:** Jennifer L. Giel, Joseph A. Sorg, Abraham L. Sonenshein, Jun Zhu

**Affiliations:** 1 Department of Microbiology, University of Pennsylvania School of Medicine, Philadelphia, Pennsylvania, United States of America; 2 Department of Molecular Biology and Microbiology, Tufts University School of Medicine, Boston, Massachusetts, United States of America; Columbia University, United States of America

## Abstract

*Clostridium difficile*, a spore-forming bacterium, causes antibiotic-associated diarrhea. In order to produce toxins and cause disease, *C. difficile* spores must germinate and grow out as vegetative cells in the host. Although a few compounds capable of germinating *C. difficile* spores in vitro have been identified, the in vivo signal(s) to which the spores respond were not previously known. Examination of intestinal and cecal extracts from untreated and antibiotic-treated mice revealed that extracts from the antibiotic-treated mice can stimulate colony formation from spores to greater levels. Treatment of these extracts with cholestyramine, a bile salt binding resin, severely decreased the ability of the extracts to stimulate colony formation from spores. This result, along with the facts that the germination factor is small, heat-stable, and water-soluble, support the idea that bile salts stimulate germination of *C. difficile* spores in vivo. All extracts able to stimulate high level of colony formation from spores had a higher proportion of primary to secondary bile salts than extracts that could not. In addition, cecal flora from antibiotic-treated mice was less able to modify the germinant taurocholate relative to flora from untreated mice, indicating that the population of bile salt modifying bacteria differed between the two groups. Taken together, these data suggest that an in vivo-produced compound, likely bile salts, stimulates colony formation from *C. difficile* spores and that levels of this compound are influenced by the commensal gastrointestinal flora.

## Introduction

*Clostridium difficile* is a spore-forming, Gram-positive bacterium estimated to be responsible for about one-quarter of hospital-acquired infections [1]. *C. difficile* causes a watery diarrhea, and transmission of this pathogen likely occurs through ingestion of *C. difficile* spores. *C. difficile*-associated disease (CDAD) can progress to intestinal lesions, resulting in pseudomembranous colitis characterized by raised yellow plaques throughout the mucosa of the colon. Though progression to toxic megacolon, intestinal perforations, peritonitis, and death is uncommon, it does occur [1], [2]. Although *C. difficile* is an obligate anaerobe when in its vegetative state, its spores have been estimated to persist on dry, inanimate surfaces for months [3], contributing to its role as a major nosocomial pathogen. In fact, *C. difficile* has been suggested to be the major infectious cause of diarrhea caused by antibiotic usage in human adults [2] and is especially a problem in elderly and immunocompromised patients. Although CDAD has a low mortality rate, *C. difficile* infection causes longer hospital stays, and treatment costs are estimated to be more than $3 billion per year in the U.S. [4]. The beginning of this century has been marked by a doubling of the rate of CDAD throughout the United States [1], and the recent emergence of hypervirulent *C. difficile* strains has resulted in higher rates of CDAD-associated morbidity, mortality, and health care costs [5], [6].

Two events usually occur prior to development of CDAD: administration of antibiotics [7], leading to disruption of commensal bacteria in the host intestine [8], and infection with *C. difficile*, likely via the spore form. Since *C. difficile* infection manifests in patients undergoing antibiotic treatment, it is not surprising that *C. difficile* is naturally resistant to a number of antibiotics [9]. Treatment has mainly relied on the antibiotics metronidazole and vancomycin [1], [10], which are unsatisfactory given that they prevent reestablishment of the commensal flora so relapses are common [1], and failure rates for metronidazole are on the rise [11], [12].

Two important aspects of *C. difficile* infection are germination of the spores and how this process is regulated in the intestinal environment. Despite the fact that *C. difficile* is likely acquired via ingestion of spores (as the vegetative form will die in the presence of oxygen), only the vegetative form produces toxins. Thus, to more fully understand pathogenesis of *C. difficile*, a better understanding of spore germination is needed. In general, bacterial spores germinate in response to the binding of one or more small molecules, and the small molecules that can cause germination vary among different species and strains of bacteria [13]. The signals to which *C. difficile* responds have not been well-characterized, but the primary bile salts cholate, taurocholate, and glycocholate have been shown to stimulate germination in vitro [14], [15], [16]. In fact, this effect of bile salts is the basis for a standard method of titering *C. difficile* spores by colony formation [16]. However, signals to which *C. difficile* spores respond in vivo have not been identified. While it has been shown that commensal microflora may inhibit *C. difficile* growth and downregulate *C. difficile* virulence gene expression [17], [18], [19], it is not known what effects normal intestinal flora have on *C. difficile* spore germination. The identification of intestinal signals that affect *C. difficile* spore germination could lead to the discovery of compounds that inhibit germination and thus CDAD. Here, we examined colony formation from *C. difficile* spores in the presence of cecal and intestinal extracts from untreated and antibiotic-treated mice as well as the ability of flora from these mice to modify the primary bile salt taurocholate.

## Materials and Methods

### Strains and Growth Conditions

*C. difficile* CD196 [20] was grown in BHI liquid medium (Bacto brain-heart infusion [BD]) or BHIS plates (BHI supplemented with yeast extract to 5 mg/ml and L-cysteine to 0.1% [w/v]) at 37°C in a Don Whitley MiniMACS anaerobic chamber (80% N_2_, 10% H_2_, 10% CO_2_). Taurocholic acid (TA) (Sigma) was added to 0.1% where indicated. Spores of CD196 were prepared as previously described [14].

To construct a strain overexpressing 7α-hydroxysteroid dehydrogenase (7α-HSDH), first the *hdhA* gene (Entrez GeneID 946151), which encodes 7α-HSDH, was PCR-amplified from the MG1655-based *E. coli* strain PK7743 [21] using primers 5′-CACTCTCATATGTTTAATTCTGACAACCTGAGAC-3′ and 5′-TCTCGAGTTAATTGAGCTCCTGTACCCCACC-3.′ This DNA fragment was restriction digested and cloned into the NdeI and XhoI sites of pET-32a. The resulting plasmid, pJG32, was transformed into *E. coli* strain BL21 to give strain JG73.

### Antibiotic Treatment and Preparation of Cecal and Intestinal Extracts

Mouse protocols were approved by the Institutional Animal Care and Use Committee at the University of Pennsylvania. CD-1 mother mice were given clindamycin hydrochloride (Spectrum Chemical) or ampicillin (Research Products International) orogastrically in two doses each of 200 mg/kg of body weight in a 20 hour period and were sacrificed 24 hours after the initial antibiotic treatment. Streptomycin sulfate (Research Products International) was added to the drinking water to a final concentration of 5 mg/ml for 24 hours as previously described [22]. Mice were sacrificed by CO_2_ asphyxiation and cervical dislocation, and the small intestine, cecum, and large intestine were harvested. Organs were weighed and homogenized in 1 ml H_2_O per mg weight, pelleted, and the supernatant was filtered through a 0.2 µm filter or boiled to sterilize. Boiling was followed by centrifugation at 16,000 ×g for 5 min., and supernatants were frozen at −20°C until use. Extracts were spotted onto BHIS plates to ensure that no colonies formed from the extracts in the absence of added spores. Where indicated, extracts were dialyzed against water in a 1 kDa membrane for 4 hours. For some experiments, cholestyramine resin (Sigma) was added to a final concentration of 50 mg/ml to small intestinal or cecal extracts or to 0.1% taurocholate, rocked for 1 hour at room temperature, and pelleted by centrifugation. The supernatant from dialysis or cholestyramine treatment was boiled to sterilize and used in colony forming unit (CFU) recovery assays as described below.

### CFU Recovery Assays

Spores were incubated anaerobically with the indicated extracts for 30 min. at 37°C, at which point dilutions were made, spread onto individual BHIS and BHIS + TA plates, and colonies enumerated after overnight growth. Spores of *C. difficile* will form colonies with extremely low efficiency on plated media in the absence of a germinant such as bile salts [14], [16]. CFU recovery is reported as the CFU/ml on BHIS plates relative to those for untreated spores spread on BHIS + TA plates.

### Determination of Bile Salt Levels

Measuring the production of NADH during the oxidation of the hydroxyl groups of bile salts by hydroxysteroid dehydrogenases (HSDHs) can be used as a method to quantify bile salts [23]. Briefly, the background absorbance at 340 nm for bile salt standards (TA, deoxycholate [DCA], or chenodeoxycholate [CDCA]) or murine cecal or intestinal extracts in 900 mM glycine/NaOH buffer, pH 9.5 and 2.65 mM NAD was measured. Reactions were initiated by addition of 3α-HSDH (Worthington) at a final concentration of 80 µg/ml, and absorbance at 340 nm was measured until it no longer increased. Total concentrations of bile salts were calculated by generating standard curves and solving the equation A_340_  =  k(CA + DCA + CDCA), where k is the extinction coefficient of NADH and CA is cholate. Quantitation of bile salts with a hydroxyl group at the C7 position (i.e., primary bile salts) were performed in a similar manner, except that supernatant from cells overexpressing 7α-HSDH was used to initiate the reaction and the equation solved was A_340_  =  k(CA + CDCA). This supernatant was prepared as follows: strain JG73 was grown to mid-log phase, at which time IPTG was added to a final concentration of 1 mM to induce overproduction of 7α-HSDH. After a 2 hour induction period, cells were pelleted and frozen at −20°C. On the day of the assay, the pellet was resuspended in 0.1 M sodium phosphate, 1 mM EDTA, pH 7 buffer, sonicated, and centrifuged for 20 min. at 6000 ×g. The supernatant was immediately used in HSDH assays.

### Incubation of Taurocholate with Small Intestinal and Cecal Contents

The contents of small intestines and ceca from freshly euthanized mice treated with or without clindamycin were removed in the anaerobic chamber and washed five times with PBS to remove any bile salts present. Pellets were resuspended in 0.1% TA and incubated anaerobically for 24 hours at 37°C. Samples were pelleted, and the supernatant was boiled to sterilize for use in CFU recovery assays.

## Results

### An Ex Vivo Factor Stimulates Colony Formation from *C. difficile* Spores

**Figure 1:**
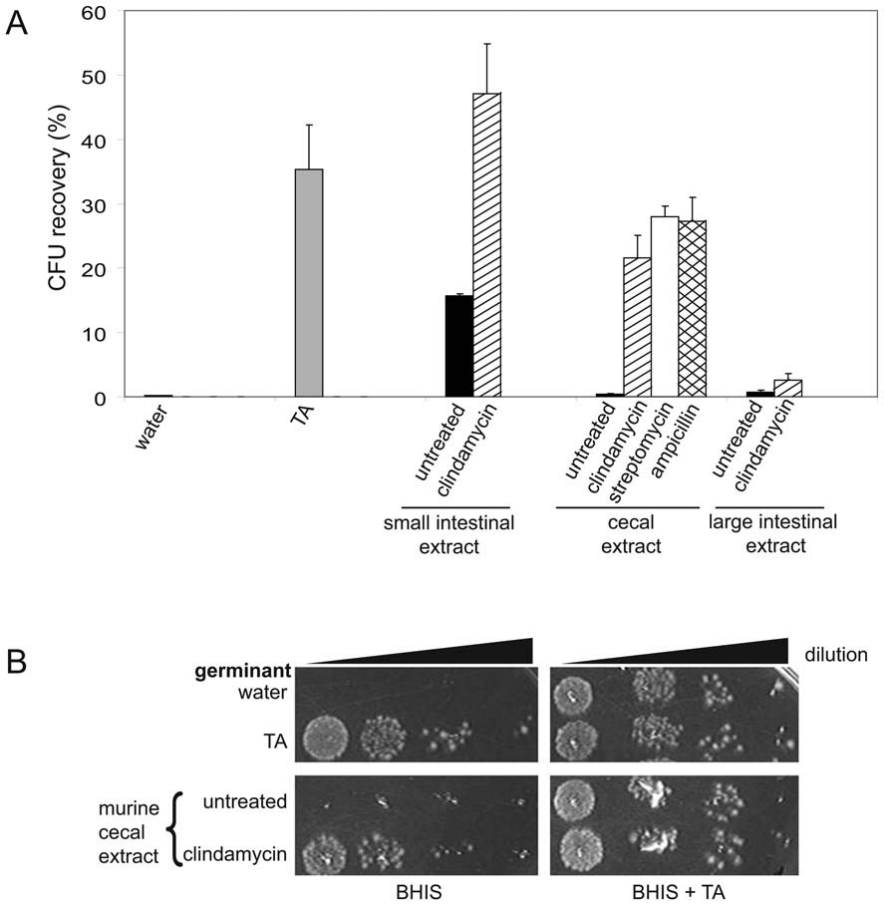
An in vivo-produced factor can stimulate CFU recovery from spores of *C. difficile*. **A**) CD196 spores were incubated for 30 min. with water, 0.1% taurocholate (TA) (gray bar), or extracts from mice that were untreated (black bars) or treated with clindamycin (striped bars), streptomycin (white bar), or ampicillin (cross-hatched bar), then diluted and spread to BHIS plates. The CFU/ml were compared to the CFU/ml on BHIS plates containing taurocholate, which was considered to reflect 100% germination. Data are the mean of at least three experiments, with error bars representing the standard error of the mean. **B**) Plates from a CFU recovery experiment in which spores were incubated with water, TA, or cecal extract from untreated or clindamycin-treated mice as in (A), then serially diluted ten-fold and spotted onto BHIS or BHIS + TA plates. A representative experiment is shown.

To examine whether compounds present in the mouse gastrointestinal tract could stimulate germination of *C. difficile* spores, separate extracts of small intestines, ceca, and large intestines from untreated adult mice were prepared and used in CFU recovery assays. The density of the extracts precluded the use of a spectrophotometric assay for germination [14]. Dormant spores have a very low efficiency of colony formation on BHIS medium unless the medium contains an appropriate bile salt; in contrast, germinated spores form colonies with high efficiency on BHIS medium [14]. Incubation of spores with cecal or large intestinal extracts led to low levels of colony formation, whereas incubation of spores with small intestinal extracts resulted in about 15% CFU recovery (Fig. 1A). These results indicate that there is a factor present in mouse small intestines that can stimulate germination of *C. difficile* spores.

### Disruption of the Commensal Flora with Antibiotics Leads to Higher Levels of Colony Formation from *C. difficile* Spores

Treatment with clindamycin and ampicillin often precipitate CDAD in humans [24], and both have been used in animal models to disrupt normal flora and induce CDAD [25], [26], so cecal extracts from mice treated with clindamycin, ampicillin, and streptomycin were also tested for their ability to stimulate colony formation from *C. difficile* spores. Cecal extracts from all of the antibiotic-treated mice showed roughly equal abilities to stimulate colony formation from *C. difficile* spores, which was about 50− to 65-fold higher than cecal extracts from the untreated mice (Fig. 1A and B). The small intestinal extracts from the clindamycin-treated mice also stimulated high levels of colony formation, but the large intestinal extract did not.

**Figure 2:**
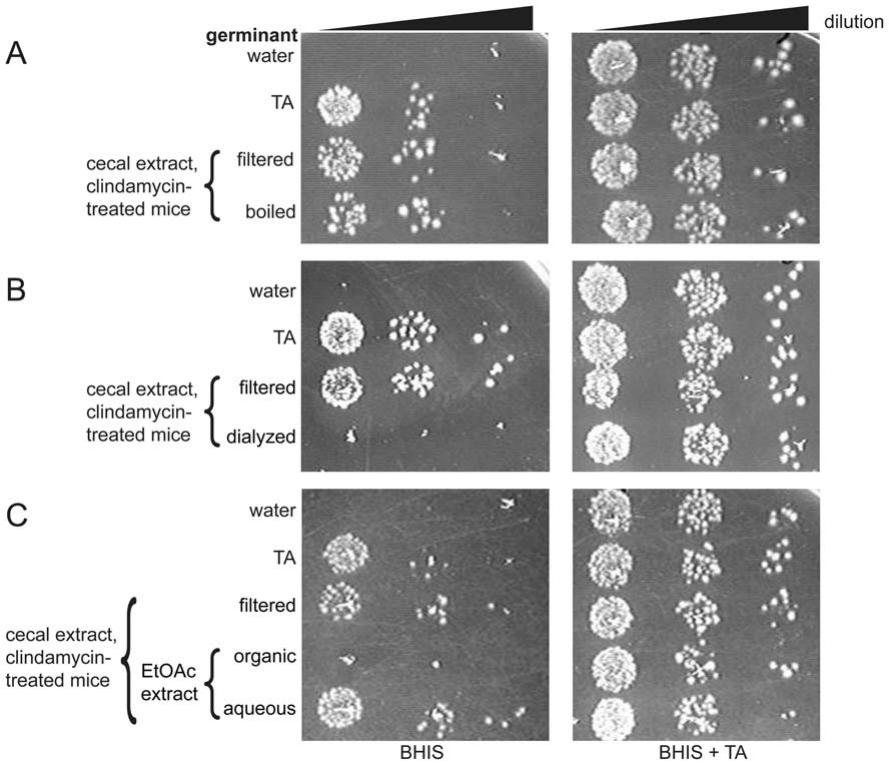
Investigation of properties of the germination factor. Cecal extracts from clindamycin-treated mice were **A**) boiled for 10 min., **B**) dialyzed against H_2_O in a 1 kDa membrane, or **C**) extracted with ethyl acetate (EtOAc) and were tested as in Fig. 1.

Since the CFU recovery assay measures not only spore germination but also outgrowth of the vegetative cells, germination efficiency was also measured by the loss of heat resistance. Briefly, spores were incubated with the cecal extracts from untreated or clindamycin-treated mice for 30 min. at 37°, heat shocked for 20 min. at 60°, then diluted and plated on BHIS + TA plates. Spores incubated with cecal extract from untreated mice were several-fold more heat-resistant than were spores incubated with cecal extract from clindamycin-treated mice (data not shown). Although the reason for the differences in magnitude between the CFU recovery assay and the heat shock experiment is unclear, it is unlikely that outgrowth was inhibited to a great degree in the CFU recovery assay. In the CFU recovery assay, spores treated with different extracts showed comparable numbers of colonies on plates containing TA (data not shown), as can be appreciated in Fig. 1B (also see Fig. 2). This would not be the case if outgrowth of the colonies were greatly inhibited. Thus, it appears that the cecal and intestinal extracts stimulate germination of the spores.

### Characterization of the Germination Factor

Several approaches were used to characterize the physical properties of the factor causing germination of *C. difficile* spores. First, to ensure that antibiotics alone did not stimulate spore germination, spores were incubated with streptomycin at a final concentration of 200 µg/ml. The CFU recovery from streptomycin-treated spores was comparable to the water control, indicating that streptomycin alone does not stimulate spore germination (data not shown). To test the heat stability of the germination factor, cecal extracts from clindamycin-treated mice were boiled for 10 min.; these extracts showed no difference in CFU recovery compared to extracts sterilized by passage through a 0.2 µm filter, indicating that the germination factor is heat-stable and can pass through a 0.2 µm filter (Fig. 2A). The germination factor appears to be small, since dialysis of cecal extract from the clindamycin-treated mice in a membrane with a 1 kDa cutoff abolished its ability to stimulate CFU recovery from spores (Fig. 2B). Testing the organic and aqueous fractions from ethyl acetate extraction indicated that the germination factor goes to the aqueous, not the organic, phase (Fig. 2C). Taken together, these data indicate that the germination factor is a compound present at higher levels in vivo in response to antibiotic treatment (but is not itself the added streptomycin), and is small, heat-stable, and water-soluble.

### Treatment of Extracts with Cholestyramine Decreases Their Ability to Stimulate Colony Formation from *C. difficile* Sspores

**Figure 3:**
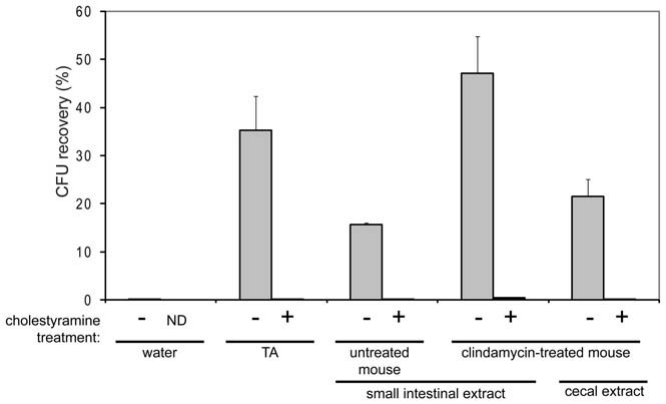
Cholestyramine treatment of small intestinal and cecal homogenates decreases their ability to induce spore germination. CD196 spores were incubated with the indicated germinant for 30 min, then diluted and spread onto BHIS plates. The CFU/ml were compared to the CFU/ml on BHIS plates containing taurocholate (TA), which was considered to reflect 100% germination. Data are the mean of at least three experiments, with error bars representing the standard error of the mean. ND, not done.

These physical characteristics and the presence of the factor in untreated and antibiotic-treated mice (albeit at different locations along the gastrointestinal tract) are consistent with bile salts as an in vivo germination factor. Previous in vitro work has shown that purified primary bile salts such as taurocholate, glycocholate, and cholate stimulate spore germination [14], [16]. As a test of whether bile salts are an in vivo germination factor, the bile acid sequestrant cholestyramine was used. Treatment of 0.1% taurocholate with cholestyramine resin decreased its ability to germinate spores about 200-fold (Fig. 3). Similarly, cholestyramine treatment of the cecal and small intestinal extracts from the clindamycin-treated mice and the small intestinal extracts from the untreated mice severely decreased their abilities to stimulate colony formation from *C. difficile* spores (Fig. 3). Spores that had been incubated with cholestyramine-treated samples were still able to germinate on plates containing TA, indicating that the resin itself did not interfere with germination. Importantly, bile salt levels in the cholestyramine-treated samples were below the limits of detection in hydroxysteroid dehydrogenase (HSDH) assays (data not shown). These results support the idea that the germination factor may be a bile salt that is present in the small intestines of untreated mice and in the cecal and small intestinal extracts of antibiotic-treated mice.

### Murine Small Intestinal and Cecal Extracts That Can Stimulate Colony Formation from Spores Contain Higher Proportions of Primary Bile Salts

**Figure 4:**
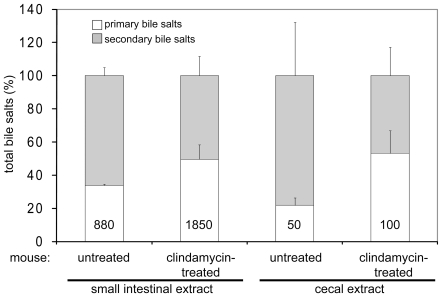
Proportions of primary bile salts in small intestinal and cecal extracts. Quantitation of bile salts in small intestinal and cecal extracts from untreated and clindamycin-treated mice was performed as previously described [23]. Data are reported as the mean percentages of total bile salts, with levels of primary bile salts (µM) indicated in the bars and error bars representing the standard error of the mean.

To examine whether bile salts levels differ among small intestinal and cecal extracts from untreated and clindamycin-treated mice, HSDH assays were carried out as previously described [23]. Total bile salt levels in extracts from small intestines were about 10-fold higher than those from cecal extracts (Fig. 4), also consistent with the greater ability of small intestinal extracts to stimulate colony formation from spores. Surprisingly, the total levels of bile salts in cecal extracts from untreated and clindamycin-treated mice were similar despite their differing abilities to stimulate CFU recovery from spores. Because some bile salts can inhibit spore germination and/or outgrowth [14], [27], additional HSDH assays were performed to quantify primary and secondary bile salts (Fig. 4). Indeed, extracts that stimulated colony formation from spores contained higher levels and proportions of primary bile salts than did an extract that stimulated colony formation much less (i.e., cecal homogenate from untreated mice).

### Treatment of Taurocholate with Small Intestinal and Cecal Flora Reduces Its Ability to Stimulate Colony Formation from *C. difficile* Spores

**Figure 5:**
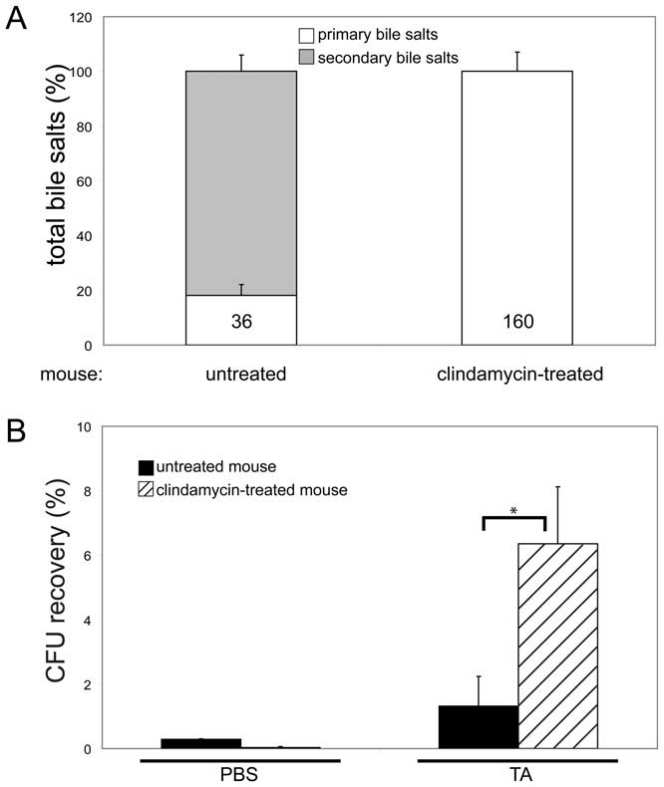
Modification of taurocholate by cecal flora and its ability to stimulate CFU recovery from spores. Contents of the ceca from freshly euthanized, clindaymin-treated (striped bars) or untreated (black bars) mice were isolated under anaerobic conditions, washed 5 times to remove any bile salts present, and incubated with 0.1% taurocholate for 24 hours at 37° C. Samples were pelleted, and the supernatant was boiled and used in **A**) bile salt quantitation and **B**) CFU recovery assays. The CFU/ml were compared to the CFU/ml recovered from spores incubated with taurocholate, which was considered 100% germination. Data are the mean of at least three experiments, with error bars representing the standard error of the mean. (* p<0.05, Student's t-test).

Several species of commensal bacteria have been shown to modify primary bile salts to secondary bile salts via 7α-dehydroxylation in vitro [28]. Secondary bile salts such as deoxycholate, formed by the deconjugation and dehydroxylation of primary bile salts by normal anaerobic intestinal bacteria, inhibit the growth of *C. difficile* [14], [15]. Since antibiotic treatment disrupts the commensal gastrointestinal flora, there may be differences in the bile salt modifying ability of bacteria from the small intestines and ceca of organisms treated with antibiotics versus those untreated. To test this point, cecal contents were anaerobically isolated from untreated and clindamycin-treated mice, washed with PBS to remove endogenous bile salts, and incubated with 0.1% taurocholate under anaerobic conditions for 24 hours at 37° C. The bile salt levels in the supernatant from this incubation were quantified (Fig. 5A), and the TA that had been incubated with cecal contents from clindamycin-treated mice had not been converted to secondary bile salts. In contrast, over 80% of the bile salts in the supernatant from incubation of TA with cecal contents from untreated mice were in the secondary form. Thus, it appears that the populations of bile salt modifying bacteria differ between untreated and antibiotic-treated mice.

To test its ability to stimulate colony formation from spores, sterilized supernatant from this incubation was used in CFU recovery assays (Fig. 5B). Taurocholate that had been incubated with cecal contents from the clindamycin-treated mice exhibited 2− to 5-fold higher levels of CFU recovery, respectively, than taurocholate incubated with contents from untreated mice. Thus, it appears that flora present in the ceca of non-antibiotic-treated mice can modify taurocholate and decrease its ability to stimulate colony formation from *C. difficile* spores.

## Discussion

Here, we have shown that colony formation from *C. difficile* spores is stimulated by an in vivo-produced factor present in the gastrointestinal tract, and that this factor appears to be present at higher levels in mice treated with antibiotics. These results are consistent with those from a recent paper in which *C. difficile* spores germinated in the presence of small intestinal and cecal contents from mice treated with a proton pump inhibitor [29]. Intriguingly, a recent study showed that clindamycin treatment of *C. difficile*-infected mice appeared to induce a highly contagious “supershedder” state, which indicates that antibiotic treatment also promotes transmission of the spores [30]. This mouse model appears to be relevant to the human disease since infection of germ-free mice with *C. difficile* resulted in large intestinal inflammation [31]. In addition, the histopathology of antibiotic-treated mice infected with *C. difficile* was similar to that of humans with CDAD, including the presence of pseudomembranous colitis [32].

The in vivo-produced germination factor may be bile salts, based on its physical properties as well as the result that treatment of extracts with cholestyramine, a bile salt sequestrant, eliminated their abilities to stimulate colony formation from *C. difficile* spores. Bile salts have been shown to have different effects in vitro: some (taurocholate, cholate, deoxycholate) stimulate germination, some inhibit germination (chenodeoxycholate), and some inhibit growth of vegetative cells (deoxycholate, chenodeoxycholate) [14], [27]. For this reason, the ratio of primary to secondary bile salts was determined, and those extracts that could stimulate colony formation from *C. difficile* spores contained higher levels of primary bile salts than those that could not. It is important to note, however, that chenodeoxycholate is a primary bile salt and we were unable to perform the 12α-HSDH assay that would allow for comparison of the cholate and chenodeoxycholate levels. We hypothesize that the extracts able to stimulate CFU recovery have low levels of chenodeoxycholate (that is, that the majority of the primary bile salts were cholate or its derivatives) because chenodeoxycholate can inhibit spore germination even in the presence of cholate and taurocholate [27]. In addition, the HSDH assays do not detect sulfated bile salts, which are present in the murine gastrointestinal tract [33], and detect muricholate, whose effect on germination is unknown, so these reported bile salt levels may be approximate.

Based on the results presented here, cholestyramine may appear to be a good candidate for treatment of CDAD. In fact, cholestyramine (Questran) has been used to treat CDAD in humans [34] and in the hamster model [25] with mixed results [35], [36]. Its mode of action was initially thought to be via toxin binding, which has been shown in vitro [37]. Perhaps the limited efficacy of cholestyramine in treating CDAD is due to the fact that bile acid synthesis increases 4− to 6-fold upon its ingestion and/or because ileal absorption of bile salts may outcompete the ability of cholestyramine to bind them [38]. In addition, cholestyramine can bind to vancomycin, one of the antibiotics used to treat CDAD [37].

We also investigated the ability of cecal flora from untreated and clindamycin-treated mice to modify the bile salt taurocholate. The results suggest that populations of bile salt modifying bacteria differ between untreated and antibiotic-treated mice, consistent with recent reports showing that antibiotic treatment of mice greatly reduces the diversity of their gastrointestinal flora [30], [39]. In addition, taurocholate that had been incubated with cecal contents from clindamycin-treated mice exhibited higher levels of CFU recovery than taurocholate incubated with contents from untreated mice. It is worth noting that the taurocholate that had been incubated with cecal contents was less able to stimulate colony formation from *C. difficile* spores compared to pure 0.1% taurocholate. Though 7α-dehydroxylation of taurocholate to deoxycholate may have occurred, the taurocholate also could have been dehydroxylated at C12 by Bacteroides species [40], resulting in conversion to chenodeoxycholate, which inhibits spore germination even when cholate or its derivatives are present, as mentioned above [27].

Intriguingly, disruption of intestinal bacteria by antibiotic treatment appears to cause differences in bile salt levels in vivo, since levels of secondary bile salts in the feces of rats treated with β-lactam antibiotics decreased while primary bile salt levels increased [41]. A similar result was observed in humans despite the bile salt differences between these two species [38]. Stools from human volunteers treated with neomycin, an aminoglycoside like streptomycin, exhibited an increase in the primary bile salt cholic acid compared to the level prior to antibiotic treatment [42] and showed a decrease in the amount of 7α-dehydroxylation activity [43], supporting the idea that changes in bile salt levels were due to a decrease in the commensal flora that carry out 7α-dehydroxylation of bile salts.

### Relationships among Antibiotic Treatment, Commensal Flora, Bile Salt Metabolism, and Germination of Spores of *C. difficile*

A model emerges in which *C. difficile* spores can be germinated by primary bile salts such as cholate and its derivatives in the small intestine and cecum. In healthy humans, CDAD may be avoided because spore germination can be inhibited by the primary bile salt chenodeoxycholate [27]. For those *C. difficile* spores that manage to germinate in response to cholate and its conjugates, outgrowth of the vegetative cells would be inhibited by the secondary bile salt deoxycholate in the large intestine, where bile salts are present in millimolar ranges [28], concentrations at which they can inhibit growth in vitro [14]. In contrast, when the host is treated with antibiotics, *C. difficile* spores are able to exploit the accompanying changes in bile salt levels. Antibiotic treatment disrupts the normal flora that carry out the 7α-dehydroxylation of bile salts, leaving a larger pool of primary bile salts such as cholate and its derivatives that can germinate the spores. There is likely also an unknown mechanism by which levels of chenodeoxycholate, a primary bile salt that inhibits spore germination and outgrowth, are concominantly lowered. Once *C. difficile* spores have germinated, the vegetative cells must be able grow out in order to produce toxins and lead to CDAD. In fact, the CFU/ml and toxin B levels from *C. difficile* strains grown in vitro in cecal contents from clindamycin- or ampicillin-treated mice were about 10,000-fold higher after 24 hours relative to those grown in cecal contents from non-antibiotic-treated mice [19], [44]. This indicates that treatment of the host with antibiotics creates a favorable environment for growth of and toxin production by *C. difficile* vegetative cells, which would lead to CDAD. The model presented here suggests that perhaps non-hepatotoxic analogs of chenodeoxycholic acid could be administered as a preventative measure to hospital patients at risk for infection with *C. difficile* or that those with the infection could be treated with antibiotic-resistant probiotics exhibiting 7α- and/or 12α-dehydroxylating activity.
